# All-optical differential equation solver with constant-coefficient tunable based on a single microring resonator

**DOI:** 10.1038/srep05581

**Published:** 2014-07-04

**Authors:** Ting Yang, Jianji Dong, Liangjun Lu, Linjie Zhou, Aoling Zheng, Xinliang Zhang, Jianping Chen

**Affiliations:** 1 Wuhan National Laboratory for Optoelectronics, Huazhong University of Science and Technology, Wuhan, China; 2 State Key Laboratory of Advanced Optical Communication Systems and Networks, Department of Electronic Engineering, Shanghai Jiao Tong University, Shanghai, China

## Abstract

Photonic integrated circuits for photonic computing open up the possibility for the realization of ultrahigh-speed and ultra wide-band signal processing with compact size and low power consumption. Differential equations model and govern fundamental physical phenomena and engineering systems in virtually any field of science and engineering, such as temperature diffusion processes, physical problems of motion subject to acceleration inputs and frictional forces, and the response of different resistor-capacitor circuits, etc. In this study, we experimentally demonstrate a feasible integrated scheme to solve first-order linear ordinary differential equation with constant-coefficient tunable based on a single silicon microring resonator. Besides, we analyze the impact of the chirp and pulse-width of input signals on the computing deviation. This device can be compatible with the electronic technology (typically complementary metal-oxide semiconductor technology), which may motivate the development of integrated photonic circuits for optical computing.

All-optical signal processing has received significant attentions in the past years in an attempt to overcome the bandwidth and speed bottlenecks incurred in conventional electronics^1^,^2^,^3^. Many optical signal processing systems, which are counterparts of the “basic functionalities” in electronic circuits, have been proposed and demonstrated over the years, including all-optical logics^4^,^5^,^6^,^7^, optical differentiation^8^,^9^,^10^,^11^,^12^,^13^,^14^ and optical integration^15^,^16^,^17^,^18^,^19^ etc. These fundamental units make more complicated optical computing functionalities come true, such as ordinary differential equation (ODE) solvers^3^,^20^,^21^. In fact, all-optical computing of a constant-coefficient linear first-order ODE can be implemented using either a first-order optical differentiator or a first-order optical integrator^3^. One of the important figure-of-merits in ODE solvers is the tunability of the constant-coefficient since changing the constant-coefficient represents a different ODE system. Although Refs. 20, 21 showed part values of constant-coefficient, the tunability was really restrained due to the lack of effective spectral shaping.

Meanwhile, silicon-based photonic integrated circuit is one of the most promising candidates for all-optical signal processing due to its intrinsic advantages of compact footprint, well integration capability and compatibility with complementary metal-oxide semiconductor (CMOS) technology. In this paper, we experimentally demonstrate an integrated all-optical system for solving first-order linear ODE with constant-coefficient tunable based on a silicon microring resonator (MRR). With different voltages applied on the MRR, the spectrum at the drop port of the MRR drifts with the quality (*Q*) factor changing, which corresponds to solving first-order linear ODE with the constant-coefficient tunable. In addition, we analyze the impact of the chirp and pulse-width of input signals on the computing deviation.

A constant-coefficient linear first-order ODE is defined as 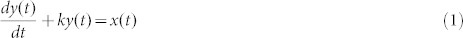
where *x*(*t*) represents the input signal, *y*(*t*) represents the equation solution (output signal) and *k* is a positive constant of an arbitrary value referred as a constant-coefficient.

In order to solve a linear first-order ODE as described in Eq. (1) in the case of zero-state response, i.e., *y*(0) = 0, the system transfer function *H*(*ω*) according to Eq. (1) should be expressed as 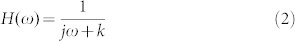
where 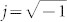, *ω* represents the optical angular frequency.

According to the time-dependent relations and coupled mode theory, the transfer function at the drop port of an add-drop MRR can be governed by^22^

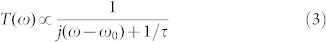
where *ω* and *ω*_0_ are the optical angular frequency and resonance frequency, *τ* represents the photon lifetime. One can see that Eq. (3) has the same formation with Eq. (2). Thus a single add-drop MRR can act as a constant-coefficient linear first-order ODE solver, where the constant-coefficient is replaced by *k* = 1/*τ*. In addition, the *Q* factor is determined by photon lifetime^23^, i.e., *Q* = *ω*_0_*τ*/2, thus we have 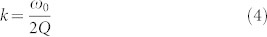
Therefore, by changing the *Q* factor of the MRR system, one can solve a linear first-order ODE with the constant-coefficient tunable. To change the *Q* factor of the MRR, Ref. 21 employed Vernier effects to randomly select several overlapped peaks of two MRRs, which was random and uncontrollable. However in this paper, we employ a MRR with carrier injection. When different voltages applied on the MRR, the carrier in the ring waveguide is changed to make the internal refractive index and loss varied. Thus the *Q* factor is continuously changed with the applied voltage.

## Results

### Device structure

**Figure 1:**
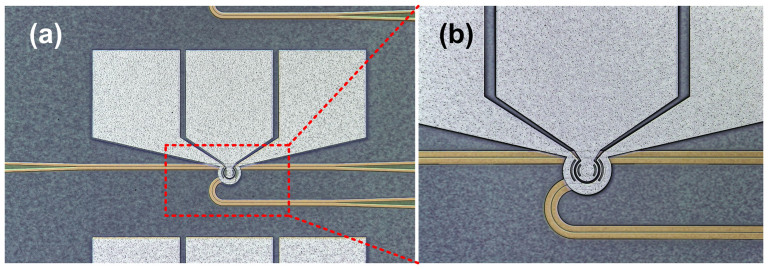
MRR design. (a) Microscope image of the fabricated MRR, (b) microscope image of the zoom-in ring region.

The add-drop MRR is fabricated on the commercial silicon-on-insulator (SOI) wafer, which consists of a ring waveguide and two straight waveguides. Figs. 1(a) and (b) show the microscope images of the fabricated MRR and the zoom-in ring region, respectively.

**Figure 2:**
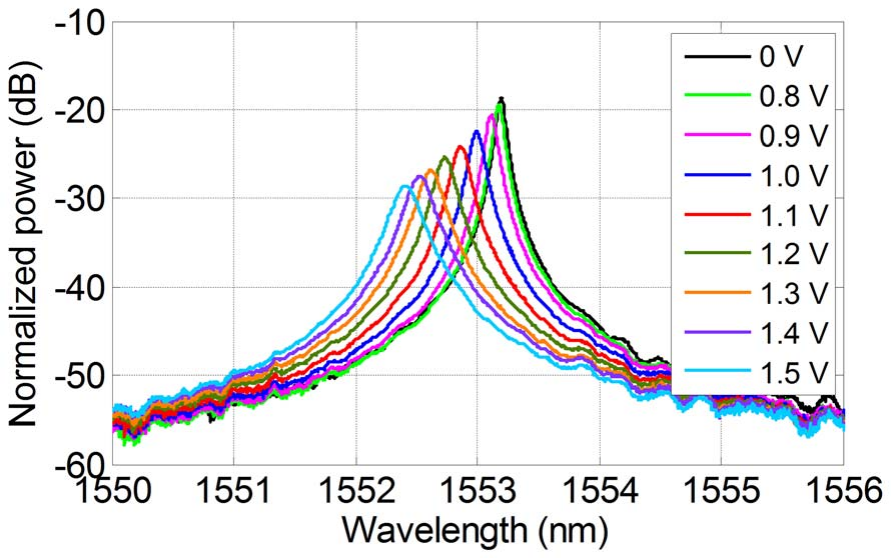
Characteristics of the MRR. Measured transmission spectra at the drop port of the fabricated MRR when varying the applied voltage.

The measured transmission spectra at the drop port of the fabricated MRR under various voltages are illustrated in Fig. 2. One can see that as the applied voltage increases, the 3-dB bandwidth is increasing, resulting in changes of *Q* factor, and the resonance frequency also experiences blue shift due to carrier dispersion effects. When 0 V voltage applied on the MRR, the 3-dB bandwidth is around 0.096 nm with a resonance wavelength of 1553.202 nm, while the applied voltage is 1.5 V, the 3-dB bandwidth becomes ~0.25 nm with a resonance wavelength of 1552.417 nm. The changes of *Q* factor result in solving first-order linear ODE with the constant-coefficient tunable.

### Experiment overview

**Figure 3:**
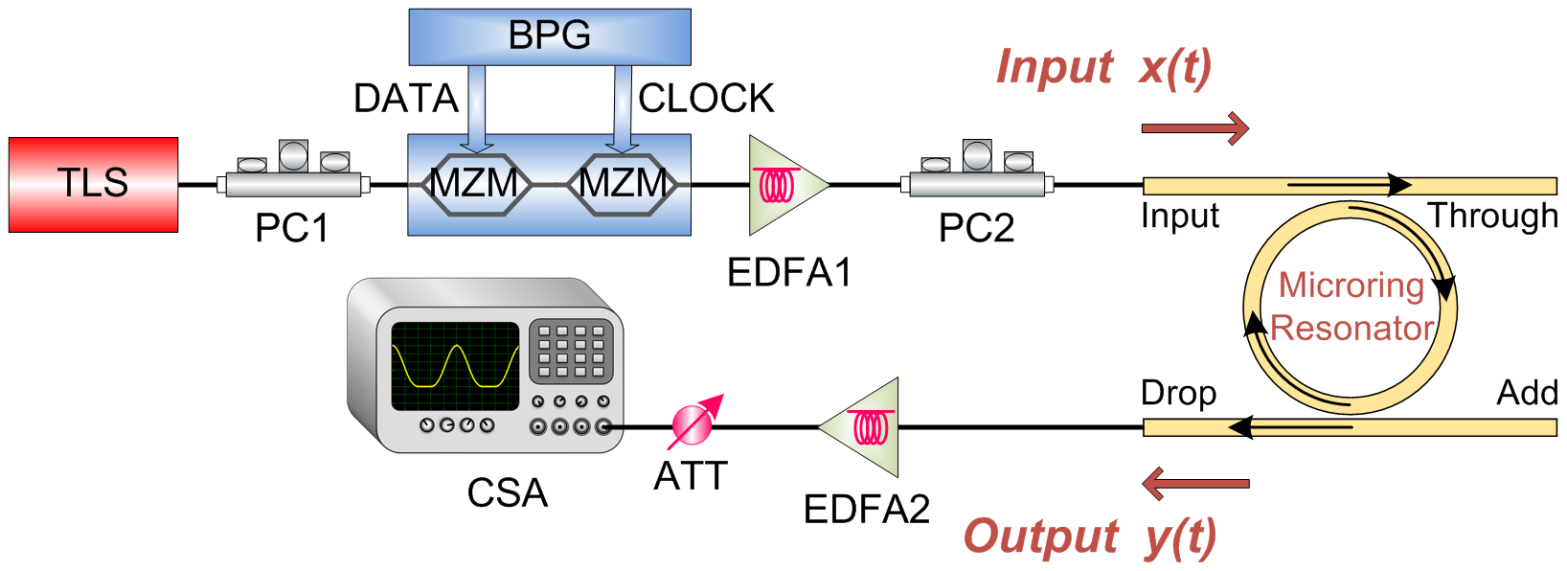
Schematic diagram of the experimental setup. First, a continuous wave beam is emitted by a tunable laser source and is then modulated by the bit pattern generator. Subsequently, the signal is coupled into the MRR by the designed grating coupler. Finally, the MRR output is amplified and observed. TLS: tunable laser source. PC: polarization controller. BPG: bit pattern generator. MZM: Mach-Zehnder modulator. EDFA: erbium doped fiber amplifier. ATT: attenuator. CSA: communication signal analyzer.

Figure 3 shows the schematic diagram for the proposed linear first-order ODE solver. A continuous wave (CW) is emitted by a tunable laser source (TLS) with a tuning resolution of 0.01 nm, which enables us to precisely align to the resonance wavelength of the MRR. And then the CW light is modulated by cascaded Mach-Zehnder modulators (MZMs) driven with self-coded data signal from a bit pattern generator (BPG). Subsequently, the generated optical signal is coupled into the MRR using vertical grating coupling method. Additionally, a direct current voltage source is used to provide different voltages applied on the MRR. Then the output signal at the drop port of the MRR is amplified by an erbium doped fiber amplifier (EDFA) and finally recorded by a communication signal analyzer (CSA). By changing the voltages applied on the MRR, we can observe different output waveforms, corresponding to ODE solution with various values of the constant-coefficient.

### Input pulse

**Figure 4:**
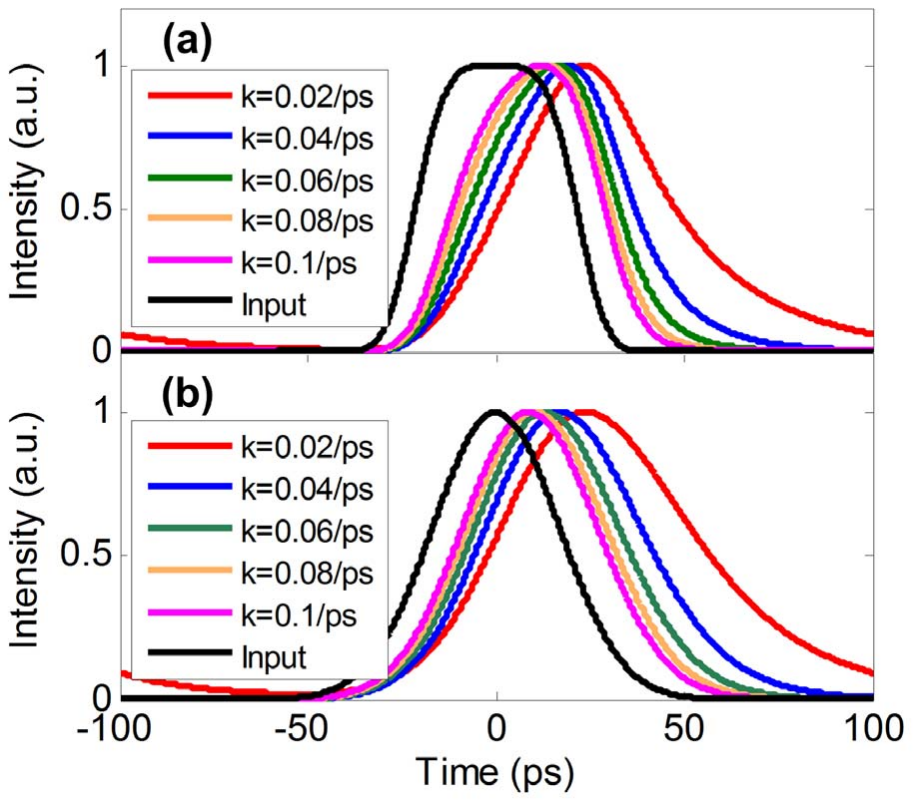
Simulated results of ODE solution. Super-Gaussian (a) and Gaussian (b) input pulses with different values of constant-coefficient.

Here super-Gaussian and Gaussian pulses are used as the input signal *x*(*t*) (as depicted in Eq. (1)). The corresponding ideal output waveforms are illustrated in Figs. 4(a) and 4(b), respectively, with different values of constant-coefficient. One can see that the output waveform is broadened when the constant-coefficient decreases. Compared to Gaussian pulse injection, the output waveform is distinctly distorted with a super-Gaussian pulse.

### Experimental results

**Figure 5:**
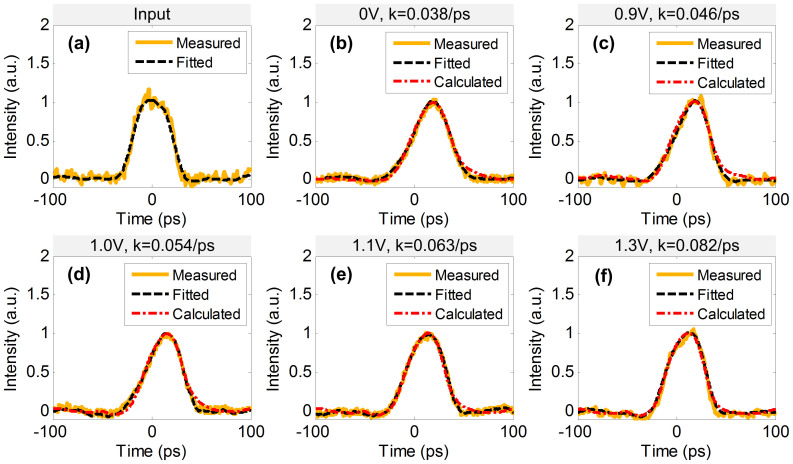
Experimental results for super-Gaussian input. (a) is the input waveform; yellow solid line: measured pulse, black dash line: fitted pulse. (b), (c), (d), (e) and (f) are the outputs with different voltages applied on the MRR; yellow solid line: measured waveforms, black dash line: fitted waveforms and red attunement line: calculated ideal outputs.

The measured input super-Gaussian waveform is depicted in Fig. 5(a) with a full width at half-maximum (FWHM) of 41.54 ps at a repetition rate of 5 GHz. When the voltage applied on the MRR is 0 V, corresponding to a constant-coefficient of about 0.038/ps, the output waveform (yellow solid line) is depicted in Fig. 5(b), and the calculated waveform (red attunement line) according to the ideal ODE solver is shown for comparison. When we change the voltages to 0.9 V, 1.0 V, 1.1 V and 1.3 V, corresponding to the constant-coefficient of 0.046/ps, 0.054/ps, 0.063/ps and 0.082/ps respectively, the measured output waveforms are depicted in Figs. 5(c), (d), (e) and (f). It should be noted that the constant-coefficient *k* is not fitted from the experimental pulse curve but calculated from the measured spectra in Fig. 2. And the calculated output waveforms are based on Eq. (2). The dash lines in Fig. 5 are the measured waveforms being smoothed by defining a moving average filter. In order to accurately evaluate the errors of ODE solutions, we need to define a parameter of average deviation, which is defined as the mean absolute deviation of measured waveforms from the calculated ones on certain pulse period (200 ps)^24^. The deviation is about 2.33%, 3.14%, 3.05%, 2.79% and 1.83% when the voltage is set at 0 V, 0.9 V, 1.0 V, 1.1 V and 1.3 V respectively.

**Figure 6:**
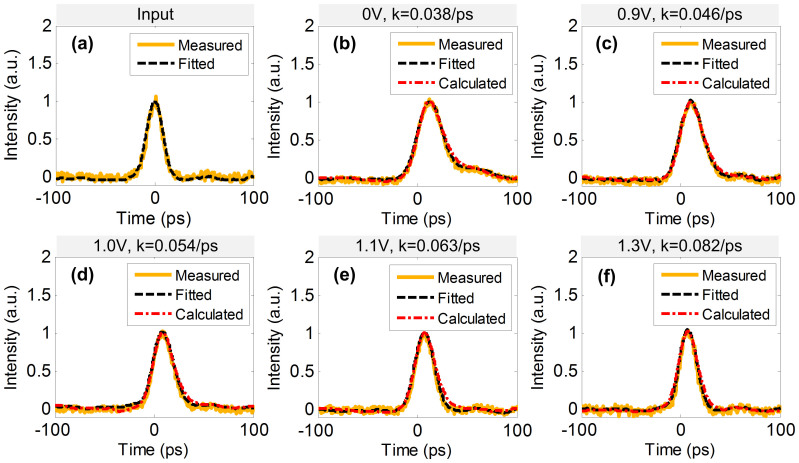
Experimental results for Gaussian input. (a) is the input waveform; yellow solid line: measured pulse, black dash line: fitted pulse. (b), (c), (d), (e) and (f) are the outputs with different voltages applied on the MRR; yellow solid line: measured waveforms, black dash line: fitted waveforms and red attunement line: calculated ideal outputs.

We then change the input signals with Gaussian pulse, and the measured input waveform is depicted in Fig. 6(a) with an FWHM of 19.07 ps at a repetition rate of 5 GHz. When we change the voltages to 0 V, 0.9 V, 1.0 V, 1.1 V and 1.3 V, corresponding to the constant-coefficient of 0.038/ps, 0.046/ps, 0.054/ps, 0.063/ps and 0.082/ps respectively, the measured output waveforms are depicted in Figs. 6(b), (c), (d), (e) and (f). And the corresponding calculated deviations are about 1.95%, 1.71%, 2.27%, 2.28% and 1.87%, respectively. For both super-Gaussian and Gaussian input pulses, one can see that all the measured waveforms fit well with the calculated ones. Thus it is feasible to use a single MRR to solve all-optical linear first-order ODE with various values of constant coefficient.

## Discussion

**Figure 7:**
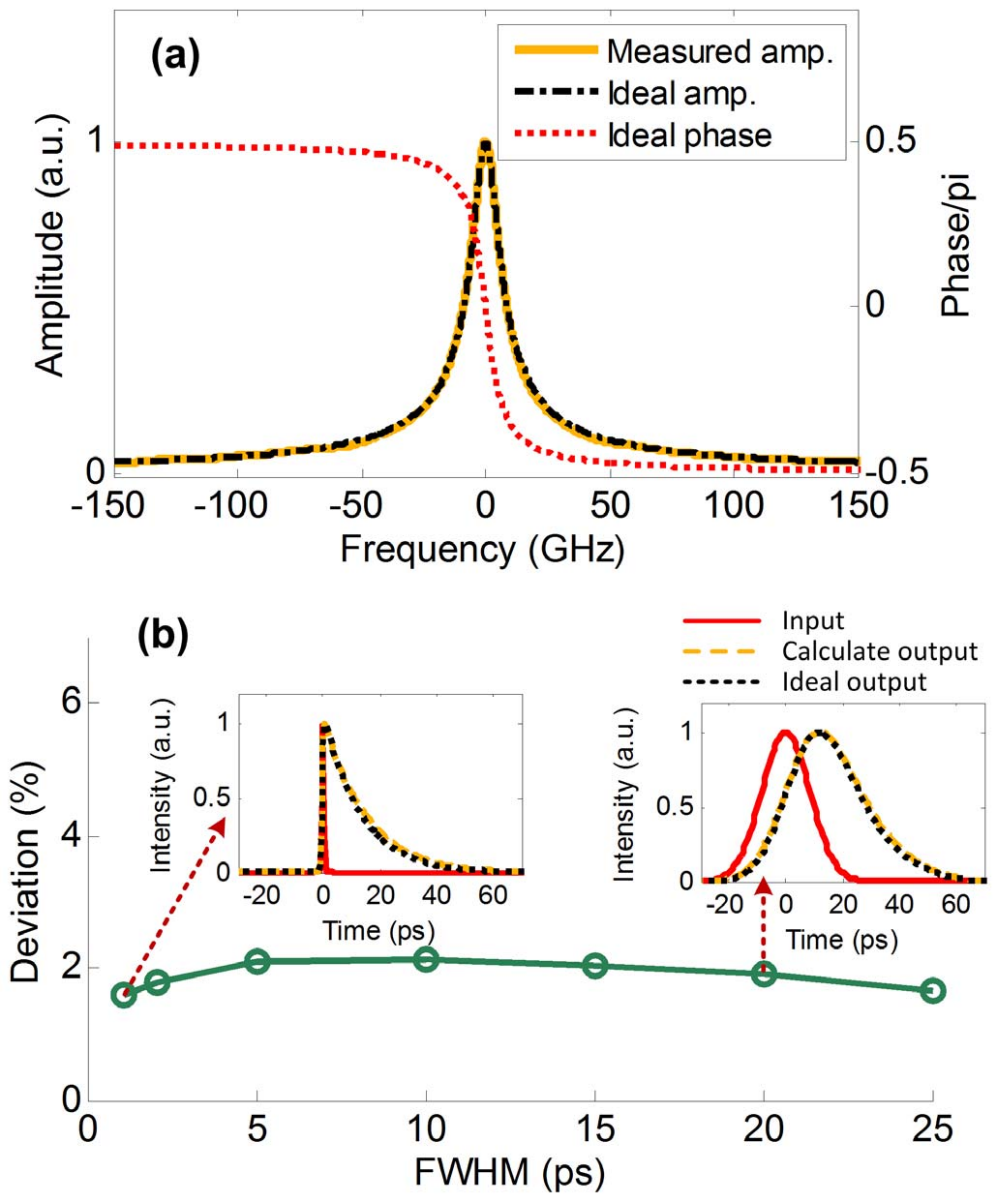
Impact of the input pulse-width. (a) yellow solid line: measured amplitude spectrum of the MRR, black attunement line: amplitude response of an ideal ODE solver, red dot line: phase response of an ideal ODE solver. (b) the average deviation as a function of input Gaussian pulse-width, insets show the input waveforms (red solid line), the calculated outputs (yellow dash line) and the ideal outputs (black dot line) with different input pulse-widths.

The transfer function of a standard MRR is a periodic frequency comb with a period fixed by the free spectral range (FSR), where the spectral response around the resonance peak can be well fitted by Lorentzian function. Figure 7(a) shows the matching between the measured amplitude spectrum (yellow solid line) of the MRR and the amplitude response of an ideal ODE solver (black attunement line, defined by Eq. (2)). The red dot line represents the ideal phase response of the ODE solver. Thus there is a good agreement between the MRR response and that of an ideal ODE solver.

To investigate the operation bandwidth of the ODE solver, we calculate the output average deviation as a function of the FWHM of input Gaussian pulses, as shown in Fig. 7(b), where the insets show the input waveforms (red solid line), the calculated outputs (yellow dash line) and the ideal outputs (black dot line) under different input pulse-widths. From Fig. 7(b), we can see the deviation is only about 2% with a wide range of pulse-widths, which indicates that the MRR may have a large operation bandwidth as an ODE solver. The operation bandwidth is only constrained by the FSR of MRR, that is, a larger FSR is corresponding to a larger processing bandwidth.

**Figure 8:**
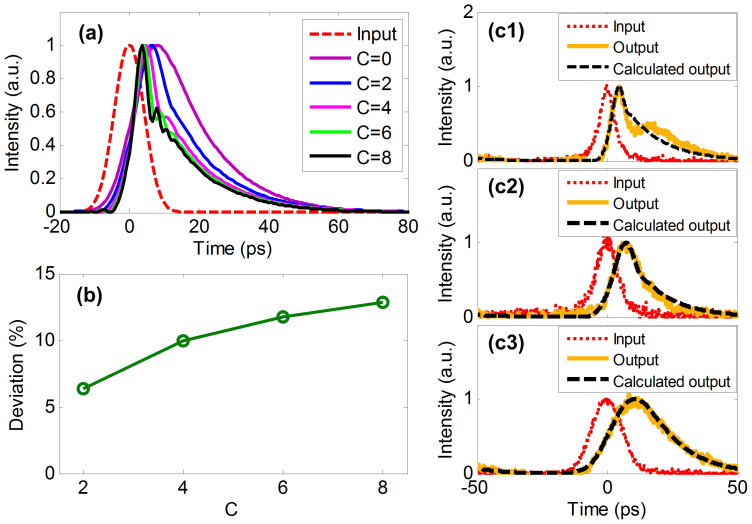
Impact of the chirped input Gaussian pulse. (a) red dash line: input pulse with fixed pulse-width, different colors of solid lines: outputs when input pulses carry different chirps. (b) the deviation as a function of the chirp parameter of the input pulse. (c1)–(c3) are the output waveforms by introducing chirp (c = 3), chirp (c = 2.5), and zero chirp (c = 0), respectively. The input pulse, measured waveform, and calculated waveform are represented by red dot line, yellow solid line, and black dash line, respectively.

Additionally, we analyze the impact of the chirped pulse on the solution deviation. For a given Gaussian pulse with a fixed FWHM of 15 ps, the output waveform of an ideal ODE solver varies with the chirp parameter *C* of the input pulse, as illustrated in Fig. 8(a). Moreover, we calculate the deviation between the output of chirped Gaussian pulse and that of non-chirped pulse, as shown in Fig. 8(b). One can see that the deviation increases as the chirp parameter increases. To experimentally verify the chirp impact, we employ another chirped pulse generator scheme (details can be found in Ref. 25). We fixed the length of the single mode fiber (SMF) following the highly nonlinear fiber (HNLF) and tuned the output power of the high power EDFA (HP-EDFA), which results in different pulse-widths and chirps of input Gaussian pulse. Figs. 8(c1)–(c3) show the input pulses (red dot line) and output waveforms of the MRR (yellow solid line), where the input pulse-width is 6.42 ps, 10.63 ps and 13.59 ps, respectively. Due to the self-phase modulation in HNLF, the chirp is larger for narrower optical pulse. The calculated output waveforms with chirps considered (black dash line) are also shown for comparison. Thus one can see that for chirped pulse input, the measured output waveforms are consistent with the calculated output waveforms with chirps considered where *C* = 3 and *C* = 2.5, as shown in Figs. 8(c1) and (c2). Therefore, our ODE solver can process complex optical signals even if it was a chirp pulse.

In summary, we demonstrate an integrated scheme capable of solving first-order linear ODE with constant-coefficient tunable based on a single MRR. With different voltages applied on the MRR, the spectrum at the drop port drifts with the *Q* factor changing, resulting in solving first-order linear ODE with different values of constant-coefficient. Additionally, we analyze the impact of the chirp and pulse-width of input signals on the computing deviation. This finding may motivate the development of integrated photonic circuits for optical computing.

## Methods

### Input waveform

For non-chirped pulse generation, we use two cascaded MZMs driven with self-coded data signal from a BPG. The pull-push MZM structure ensures non-chirp pulse generation. If both two MZMs work, the output signals are Gaussian shape. If only one MZM works, the output signals are super-Gaussian shape. For the chirped pulse generation, we use an intensity modulator and a phase modulator to generate a direct-current free Gaussian pulse, followed by a segment of SMF, an HP-EDFA, a HNLF and another segment of SMF. The phase modulator and HNLF have contributions to the chirp of output signals. The pulse-width of output signal is controlled by the power of HP-EDFA. Details can be found in Ref. 25.

### Devices fabrication

We employ a single add-drop MRR to solve first-order linear ODE. First we design and fabricate the MRR on an SOI wafer. The thickness of the top silicon and the buried oxide layer of the SOI wafer are 220 nm and 2 μm, respectively. To define the waveguide patterns, deep ultra-violet (DUV) photolithography using a 248 nm stepper was employed, followed by anisotropic dry etch of silicon. Boron and phosphorus ion implantations were performed to form the highly *p*-type and *n*-type doped regions. Also the slab layer was etched outside the *p-i-n* junctions to confine the current flow around the ring waveguide. Finally, contact holes were etched and aluminum was deposited to form the metal connection. We use vertical grating coupling method to couple the fiber and the silicon MRR^26^,^27^. The whole fabrication process is done using CMOS compatible processes.

## Author Contributions

J.J.D., T.Y. and X.L.Z. conceived the study. T.Y. and J.J.D. performed the numerical simulation. L.J.L., L.J.Z. and J.P.C. prepared the samples. T.Y. and A.L.Z. carried out the experiment. T.Y. analyzed the data and wrote the manuscript. J.J.D. supervised the project and edited the manuscript. All authors discussed the results and commented on the manuscript.
